# Genome methylation patterns across castes and generations in a parasitoid wasp

**DOI:** 10.1002/ece3.2395

**Published:** 2016-09-30

**Authors:** Roei Shaham, Rachel Ben‐Shlomo, Uzi Motro, Tamar Keasar

**Affiliations:** ^1^ Evolutionary and Environmental BiologyUniversity of HaifaHaifaIsrael; ^2^ Biology and the EnvironmentUniversity of Haifa – OranimTivonIsrael; ^3^ Evolution, Ecology and BehaviorThe Hebrew University of JerusalemJerusalemIsrael

**Keywords:** Genomic imprinting, methylation‐sensitive amplified fragment length polymorphism, polyembryony, transgenerational epigenetic effect

## Abstract

Environmental influences shape phenotypes within and across generations, often through DNA methylations that modify gene expression. Methylations were proposed to mediate caste and task allocation in some eusocial insects, but how an insect's environment affects DNA methylation in its offspring is yet unknown. We characterized parental effects on methylation profiles in the polyembryonic parasitoid wasp *Copidosoma koehleri*, as well as methylation patterns associated with its simple caste system. We used methylation‐sensitive amplified fragment length polymorphism (MS‐AFLP) to compare methylation patterns, among (1) reproductive and soldier larvae; and (2) offspring (larvae, pupae, and adults) of wasps that were reared at either high or low larval density and mated in the four possible combinations. Methylation frequencies were similar across castes, but the profiles of methylated fragments differed significantly. Parental rearing density did not affect methylation frequencies in the offspring at any developmental stage. Principal coordinate analysis indicated no significant differences in methylation profiles among the four crossbreeding groups and the three developmental stages. Nevertheless, a clustering analysis, performed on a subset of the fragments, revealed similar methylation patterns in larvae, pupae, and adults in two of the four parental crosses. Nine fragments were methylated at two cytosine sites in all larvae, and five others were methylated at two sites in all adults. Thus, DNA methylations correlate with within‐generation phenotypic plasticity due to caste. However, their association with developmental stage and with transgenerational epigenetic effects is not clearly supported.

## Introduction

How multiple phenotypes arise from a single genotype is a key issue in evolutionary and developmental biology. Mechanistically, this requires chemical modifications of DNA segments, while the nucleotide sequence remains unchanged. The biochemical mechanisms involved range from covalent and noncovalent modifications of DNA and histone proteins to RNA‐related pathways. These modifications exert epigenetic control that suppresses or activates gene expression (Smith et al. 2012).

One of the best‐characterized epigenetic mechanisms is DNA methylation. It occurs on cytosine residues of CpG dinucleotides, correlates with transcriptional repression, and plays an important role in gene regulation and chromatin organization (Goll and Bestor 2005). The enzymatic pathways that lead to DNA methylation involve multiple DNA methyltransferase proteins, including two major enzymatic complexes: DNA methyltransferase 1 (DNMT1), which is primarily responsible for maintaining previously extant DNA methylation, and DNA methyltransferase 3 (DNMT3) that establishes de novo DNA methylation (Klose and Bird 2006). In insects, DNA is not globally methylated and the importance of methylation varies greatly among taxa. For example of this variation, see Marhold et al. (2004) and Boffelli et al. (2014) on *Drosophila*, Zemach et al. (2010) on flour beetles, and Walsh et al. (2010) on pea aphids. Methylation in insects tends to target specific genes and plays an important role in the regulation of transcription and possibly RNA splicing (Glastad et al. 2011).

Similar to other insects, hymenopterans also exhibit a wide range of methylation possibilities. A full methylation toolkit was found in ants (Bonasio et al. 2012), in honeybees (*Apis mellifera,* Lyko et al. 2010), in two species of bumblebees (Sadd et al. 2015), and in a number of wasp species (Park et al. 2011). DNA methylation is potentially important in shaping phenotypes and adaptations to the environment, such as in caste determination in eusocial species (Smith et al. 2012; ‏Yan et al. 2014; but see Libbrecht et al. (2016) for a recent counter‐example). Experiments with social bees provide evidence for this hypothesis: In honeybees (*Apis mellifera*), queen larvae have lower genomewide methylation levels than worker larvae. Knockdown of DNMT3 using RNA interference (RNAi) in worker larvae induces the development of queen‐like ovaries in adults (Kucharski et al. 2008; Lyko et al. 2010). Further, chemical inhibition of DNA methyltransferase activity promotes worker reproduction in queenless bumblebee colonies (Amarasinghe et al. 2014).

Even within a caste, individuals may have different tasks, such as workers that perform household tasks, nest guarding, or foraging. This task allocation also involves modification of methylation patterns in some social hymenoptera (‏Yan et al. 2014), including two species of ants (Bonasio et al. 2012) and honeybees (Herb et al. 2012). The importance of DNA methylations was also demonstrated in a solitary hymenopteran, the parasitoid wasp *Nasonia vitripennis*. DNA methylation is prevalent in the *N. vitripennis* genome, mainly in exons, and is correlated with elevated gene expression and with genes that are expressed in all developmental stages (Park et al. 2011; Wang et al. 2013).

The studies described so far focused on *within‐generation* phenotypic variability in Hymenoptera. In addition, environmental conditions encountered by individuals also interact with their progeny's genes and environment and epigenetically affect offspring phenotypes. Such *transgenerational* parental effects are widespread and affect important life‐history traits, such as offspring survival, growth rates, adult size, diapause, dispersal, and fecundity (Jann and Ward 1999; Alekseev and Lampert 2001; Mondor et al. 2004; Roff and Sokolovska 2004). They have been described in vertebrates (Bernardo 1996), as well as in several groups of insects (Grech et al. 2007; Allen et al. 2008). DNA methylation was shown to mediate transgenerational epigenetic effects in plants, but its importance in some animals is still being discussed (Heard and Martienssen 2014). In Hymenoptera, in particular, the role of DNA methylation in transgenerational epigenetic effects has hardly been studied. So far, evidence for maternal epigenetic effects via methylation in hymenopterans comes only from the parasitoid *Nasonia vitripennis,* where all DNMT1 and DNMT3 mRNAs are maternally provided to the embryo. Lowering of the maternal DNMT1 mRNA level results in embryonic lethality (Zwier et al. 2012). This provides a potential mechanism for mothers to regulate methylation levels in their offspring, and thereby epigenetically affect their phenotype. This possibility has not yet been directly tested in Hymenoptera. This study aimed to address this knowledge gap by manipulating the rearing conditions of parasitoids and comparing among the DNA methylation profiles of their offspring.

**Figure 1:**
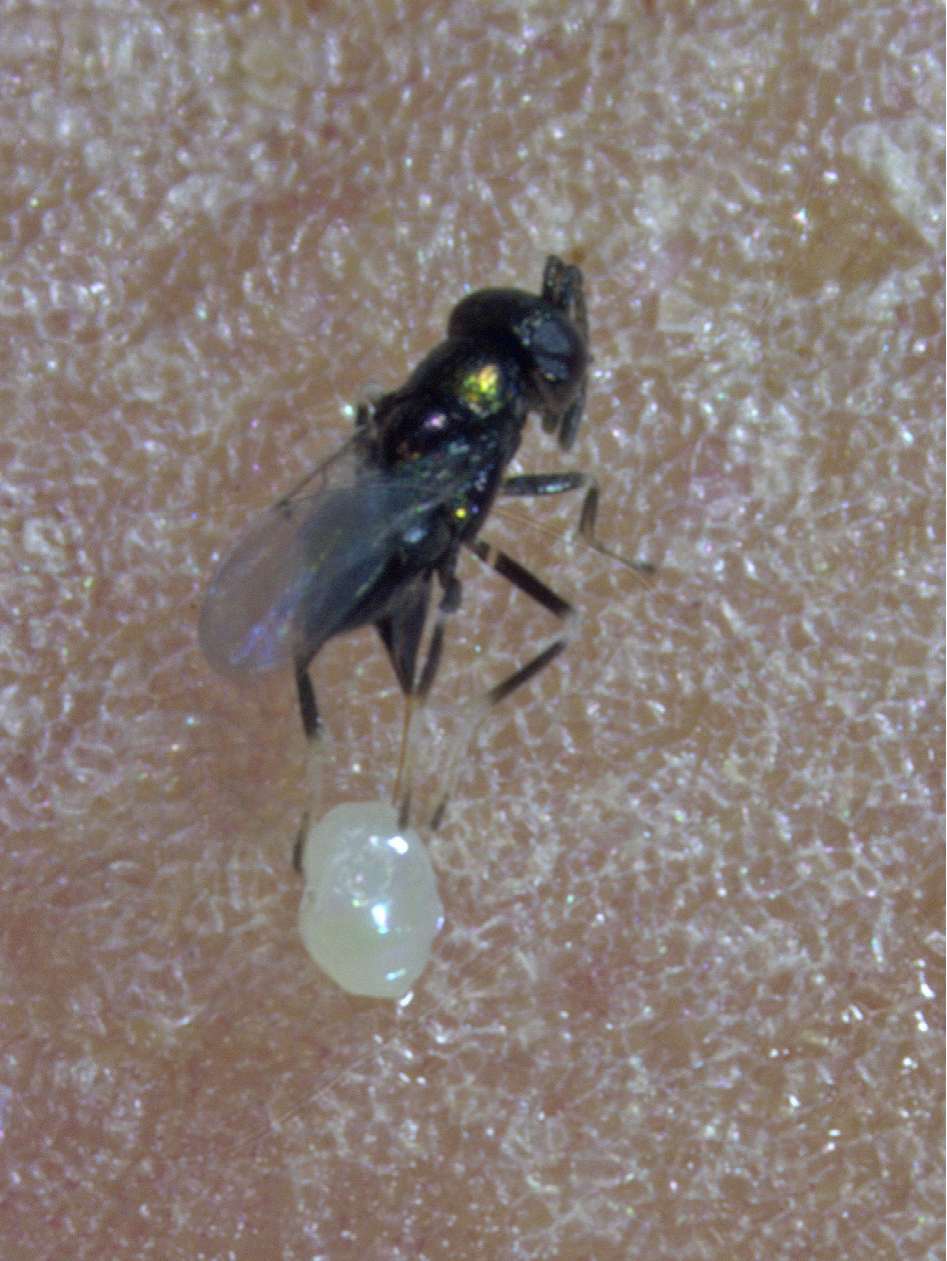
The study organism, the parasitoid wasp *Copidosoma koehleri* (Hymenoptera: Encyrtidae). Adults wasps recently emerged from their host's mummy. Photograph by Nitzan Cohen and Miriam Kishinevsky.

The life history of our study species, the encyrtid wasp *Copidosoma koehleri* (Hymenoptera: Encyrtidae, Fig. 1), provides unique opportunities to study between‐ and within‐generation epigenetic effects. *C. koehleri* females oviposit into eggs of their host, the moth *Phthorimaea operculella* (Lepidoptera: Gelechiidae). Each wasp egg proliferates into a polyembryonic mass (polymorula) that develops into a clone comprising about 40 genetically identical individuals. Like other hymenopterans, *C. koehleri* has a haplo‐diploid sex determination mechanism, in which males develop from unfertilized eggs, while females develop from fertilized ones. In female clones, one larva develops earlier than its clone mates and becomes a soldier, which does not reproduce. A soldier acts when the host contains more than one *C. koehleri* clone as a result of superparasitism (two or more eggs laid in the same host). It attacks and kills larvae of competing clones. The parasitized tuber moth larva hatches from the egg and goes through four larval instars, while the parasitoid embryos develop inside it. Eventually, the parasitoid larvae consume the host tissues until only its cuticle remains, pupate in the host mummy, and emerge as adult broods (defined as all individuals that emerge from the same host; Doutt 1947).

By exposing clone mates to different environments and characterizing methylation patterns in their offspring, we minimized influences of genetic variability. As in other parasitoids, parental care in *C. koehleri* is limited to host selection; hence, the environmental conditions experienced by parents and offspring can be independently manipulated. We have previously demonstrated that parental rearing conditions influence offspring phenotype in *C. koehleri*: The rearing density of mothers affects their offspring's rate of development (Morag et al. 2011a). Maternal mating status and host encounter rates influence the number of individuals in a clone (Morag et al. 2011b,c).

To test for effects of parental environmental exposure on DNA methylation in offspring, we compared methylation profiles among individuals whose parents developed under either high or low larval densities (HD/LD treatments). We also tested for associations between methylation and within‐generation variability, namely across developmental stages and between the two reproductive castes of *C. koehleri*.

## Materials and Methods

### Laboratory conditions

A laboratory stock of *Copidosoma koehleri*, originating from a field collection in South Africa in 2003, was used. Parasitoids and their hosts were reared at 25 ± 3°C, 40% humidity, and a 12:12‐h L:D illumination cycle. The same laboratory conditions were maintained during experiments. *Phthorimaea operculella* hosts were provided with potatoes as food during their four larval instars. Adult *C. koehleri* fed on honey. The rearing densities of hosts and parasitoids in the insectary culture varied over the period of the study and were not controlled.

### Experiment 1 – Methylation in reproductive and soldier larvae

One male clone and one female clone from the insectary colony (each of them containing about 40 genetically identical wasps) were placed together and allowed to mate for 48 h. This was carried out in ten replicates, each of which contained a single maternal and a single paternal genotype. Subsequently, 200–300 fresh eggs of *P. operculella* were added to each replicate. These hosts were available for parasitism by the mated *C. koehleri* females for 48 additional hours. The hosts were reared on potatoes for 7–10 days. Third‐instar *P. operculella* parasitized larvae were collected, and the wasp larvae were dissected out of these hosts into insect Ringer solution. All wasp larvae within a replicate were genetically highly related, because both of their parents were clone mates originated from an inbred laboratory stock. The reproductive females from all hosts, within each of the ten replicates, were collected into one vial, and all the soldiers were collected into a second one. This ensured that enough wasp tissue of closely related individuals was available for the genetic analysis, as was confirmed in preliminary experiments. The parasitoid larvae were kept frozen at −18°C until DNA extraction and MS‐AFLP analysis. One sample per clone and caste was analyzed.

### Experiment 2 – Parental effects on offspring methylation across developmental stages

**Figure 2:**
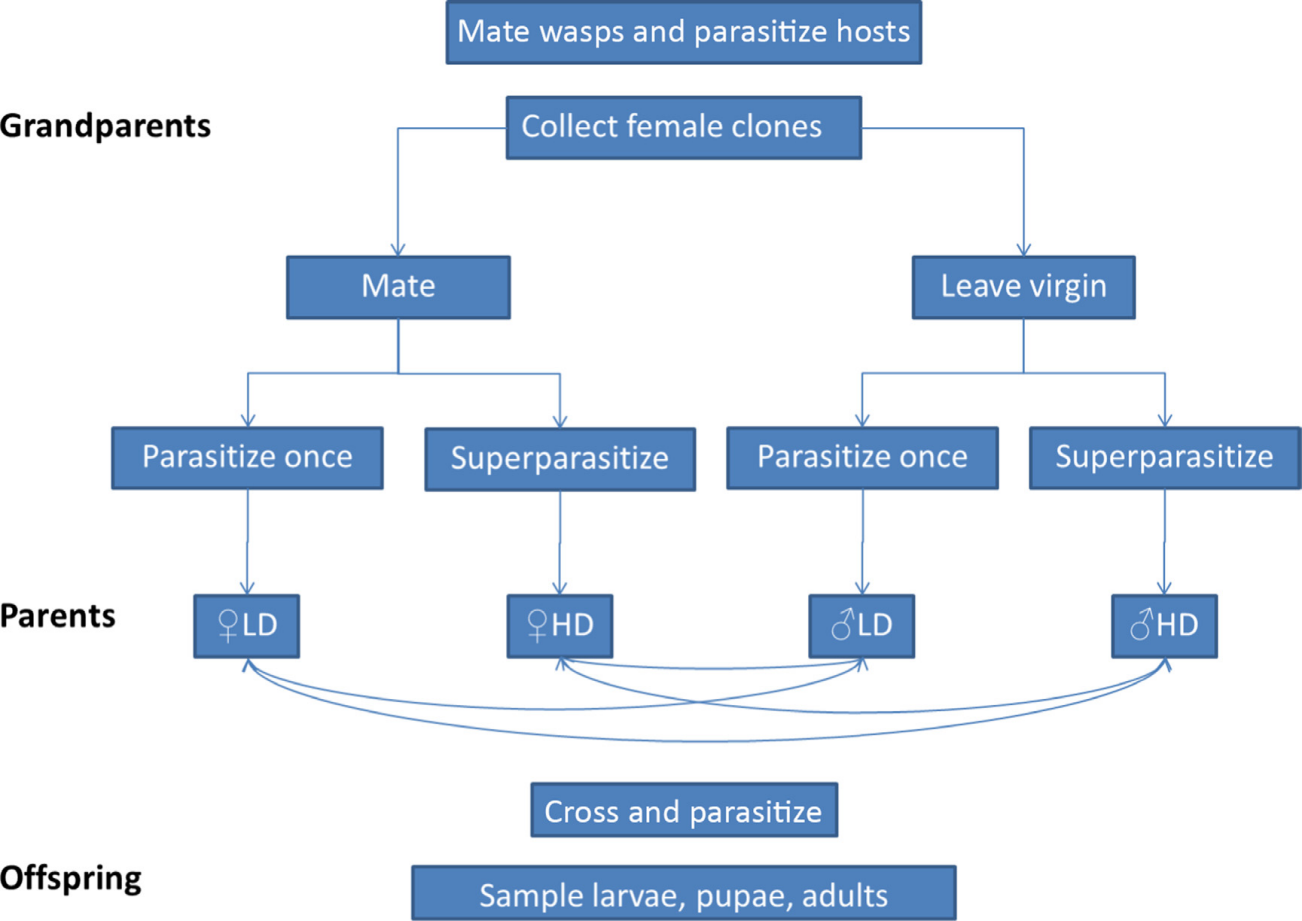
Design of Experiment 2. The number of replicates in each experimental group is reported in Table 1.

This experiment compared methylation patterns among *C. koehleri* larvae, pupae, and adults and determined whether the methylation profiles are affected by their parents' developmental history. Briefly, males and females in the parent generation were reared at either low density (LD) or high density (HD) as larvae. After they emerged from the hosts, they were mated in the four possible combinations (LD × LD, LD × HD, HD × LD, HD × HD). Their offspring were sampled as larvae, pupae, and adults. The following protocol was used (Fig. 2):

*The grandparental generation*: For each of 10 replicates, six adult clones of virgin females and three male clones were collected from the insectary culture. Each clone consisted of ca. 40 genetically identical wasps. The clones used for Experiment 2 were different from those used for Experiment 1. Three of the female clones were mated with males for 48 h as detailed in Experiment 1. The remaining three female clones remained virgin to produce the males of the next generation. At the end of the mating period, 15 females were sampled from each clone. Ten of them were housed in pairs, and the other five were housed singly. This generated 15 replicates (five replicates of each of three clones) of each of the four following parental groups: two mated females, one mated female, two virgin females, and one virgin female. A total of 20–30 fresh hosts were added to each replicate for 48 h. According to a previous experiment (Kishinevsky and Keasar 2015), pairs of females are likely to oviposit in the same hosts, resulting in superparasitism and high density (HD) conditions during the offspring's larval development. Single females, on the other hand, are likely to oviposit only once in each host, resulting in a lower density (LD) for the developing F1 larvae.
*The parental generation*: The hosts were reared on potatoes until pupation of the F1 generation larvae, which coincided with the death of the hosts. Each host mummy, containing *C. koehleri* pupae, was reared separately until adult emergence. After emergence of the F1 adults, male and female clones, which had been exposed to different densities in the larval stage, were selected for mating. For each replicate, five males and five females from each clone were paired, in the four possible combinations: (1) ♀LD × ♂HD; (2) ♀HD × ♂LD; (3) ♀HD × ♂HD; and (4) ♀LD × ♂LD. Thus, replicates differed in genetic composition from each other. After a 48‐h mating period, 20–30 hosts were added for 48 additional hours. Subsequently, hosts eggs were moved to potatoes for rearing of the F2 generation.
*The offspring generation*: Offspring female wasps were sampled from each potato at three time points during development: (1) 14 days after parasitism to extract larvae; (2) one week after pupation; and (3) after emergence of adults. Female larval broods were identified by the presence of soldier individuals. Female pupae were identified by extracting the desired samples from the pupal brood and storing them deep‐frozen, while letting the other pupae emerge as adults and sexing them. As in Experiment 1, several individuals parented by clone‐mate mothers and clone‐mate fathers were pooled to ensure sufficient tissue in each sample.

Table 1 lists the sample sizes obtained for the different mating combinations and developmental stages.

**Table 1:** Number of samples from each crossbreeding group in the larval, pupal, and adult life stages. Each sample contained whole‐body tissue from several females, whose mothers originated from a single clone and whose fathers originated from a different clone

Crossbreeding group	Larval stage	Pupal stage	Adult stage
♀LD × ♂LD	7	9	8
♀LD × ♂HD	9	9	9
♀HD × ♂LD	8	10	8
♀HD × ♂HD	8	10	9

HD, high density; LD, low density.

### MS‐AFLP‐based detection of DNA methylation

Host larvae that were collected from potato tubers were washed in water. Wasp larvae dissected out of the hosts were rinsed in water and in Ringer's solution, to reduce the risk of contamination by potato DNA. DNA was isolated from the whole bodies of the sacrificed wasps using QIAGEN^®^'s (Qiagen, Hilden, Germany) Blood & Tissue Kit following the recommended protocol. MS‐AFLP detection of DNA methylation was carried out essentially as described by Xu et al. (2000). The method uses the restriction isoschizomers *Hpa*II and *Msp*I that recognize the sequence CCGG and differ in their ability to cut methylated cytosine residues, that is, having different methylation sensitivities (Fulneček and Kovařík 2014). It is a marker‐type technique that does not accurately quantify DNA methylation, but provides insight about differences among groups in patterns of cytosine methylation based on a moderate number of anonymous loci.

The genomic DNA (~200 ng) was digested in two parallel reactions with a pair of restriction enzymes (*Eco*RI/*Msp*I and *Eco*RI/*Hpa*II) at 37°C for 5 h and then ligated to double‐stranded *Eco*RI (E‐) and isoschizomers (M‐) adaptors. The resulting fragments were pre‐amplified with nonselective primers, where the ligated adaptors served as target sites for primer annealing. Four selective primer combinations were used for AFLP amplification: E‐ACA/M‐TA, E‐ACG/M‐CT, E‐ACT/M‐CA, and E‐ACC/M‐GA (E‐ and M‐ representing the restriction site and its ligated adaptor sequence). The selective *Eco*RI (E‐) primers were labeled with florescent dyes (6‐Fam, Ned, Vic, and Pet, respectively).

PCRs were carried out in a total volume of 13 *μ*L. PCR amplification cycles started at an annealing temperature of 65°C, after which the annealing temperature was lowered by 0.7°C per cycle for 12 cycles (a touchdown phase of 13 cycles), followed by 23 cycles at an annealing temperature of 56°C. Amplification products were separated using a sequencer (ABI 3130xl Florescence‐Reader, Applied Biosystems, Darmstadt, Germany). Fragment analyses and genotyping were determined manually, directly from the chromatographs, using Peak Scanner software (Applied Biosystems) with its default settings.

DNA of ~10% of the samples was amplified and run in duplicate to validate integrity of amplifications. Samples with large numbers of detected loci were selected for this repeatability analysis.

### Data analysis

Only fragments of >80 bp were analyzed, so as to reduce the potential impact of size homoplasy (i.e., nonhomologous alleles of equal mobility). We used a more permissive cutoff value than the frequently applied threshold of 150 bp, because the number of loci detected in our samples was relatively low (see Results section), and the bands within the >80 bp region were not too dense. This reduces the potential biases due to homoplasy (Caballero et al. 2008). The collected data were entered into a binary data matrix that indicated the banding pattern generated by the isoschizomer endonucleases *Hpa*II and *Msp*I. This matrix listed the samples in rows and the DNA fragment lengths in columns. We scored each element (i, j) of the matrix as 1 if fragment j was present in sample i, and as 0 otherwise.

Using the binary data matrix, we identified and counted the methylated fragments in each sample (i.e., loci that appeared in only one of the isoschizomers: *Hpa*II+/*Msp*I− or *Hpa*II−/*Msp*I+). Fragments that were recognized by both isoschizomers (*Hpa*II+/*Msp*I+) were scored as nonmethylated loci. The R (version 3.1.2) package *Msap* (Pérez‐Figueroa 2013) was used to detect methylation‐susceptible loci (**MSL**) and nonmethylated loci (**NML**). MSL were defined as fragments that were methylated in at least 5% of the samples. Wilcoxon rank‐sum tests with continuity correction were conducted to compare diversity between MSL (diversity due to differences in methylation, that is, epigenetic diversity) and NML (diversity due to DNA sequence differences, that is, genetic diversity). The amount of genetic and epigenetic variation was estimated by the software using the Shannon diversity index (S), calculated for each locus by the formula S = −ΣPi lnPi. Pi is the frequency of the presence or absence of a fragment. The mean diversity was estimated by an average of index values over individual loci. AMOVA (analysis of molecular variance) tests were used to compare the variance within and among castes (Experiment 1), developmental stages, and crossbreeding groups (Experiment 2). Principal coordinate analysis was employed to cluster and compare soldiers and reproductive female castes and the different crossbreeding groups. These analyses are provided by the *Msap* software.

We analyzed the patterns of methylated fragments in Experiment 1 manually as well, by listing and comparing the methylated fragments found in the soldier caste and in the reproductive females' caste. Twenty‐seven fragments were methylated in four or more samples; that is, their methylation frequencies were high enough to allow testing for dependence between caste and methylation rate. For each of these fragments, we tested for independence between caste and the proportion of methylated samples using Fisher's exact test.

We also performed an additional manual analysis of the data from Experiment 2. We identified 31 loci (fragments) that were not digested by either of the endonucleases (*Hpa*II‐/*Msp*I‐) across all samples of one of the four crossbreeding groups. We adjusted the *Msap* software settings to interpret these fragments as being hypermethylated, that is, carrying methylation marks on both DNA strands and/or in both cytosine residues (Pérez‐Figueroa 2013). We listed the proportions of hypermethylated samples in each of the 12 populations (four crossbreeding groups × three developmental stages), separately for each of these fragments. This resulted in 12 arrays, one for each experimental population, whose 31 components are the proportions of hypermethylated fragments. We used Ward's method (squared Euclidean distances and minimum variance amalgamation) to calculate the similarities among these arrays and to cluster them accordingly, using the Multi‐Variate Statistical Package (version 3.22) by Kovach Computing Services, Pentraeth, Wales, UK.

Finally, we manually listed fragments that were hypermethylated across all samples in one or more of the three developmental stages, regardless of crossbreeding group. This was carried out to identify any stage‐specific methylation pattern.

IBM, Boston, Mass, USA 21 was used for the remaining statistical analyses.

## Results

### Experiment 1 – Methylation in reproductive and soldier larvae

Seven paired samples of soldiers and reproductive females, of the ten replicates in the experiment, generated sufficient data for analyses. In total, three primer pairs (E‐ACG/M‐CT, E‐ACT/M‐CA, and E‐ACC/M‐GA) yielded 128 fragments that were methylated in at least two samples. Repeatability levels were 95.01% for methylated fragments and 99.5% for nonmethylated ones.

**Figure 3:**
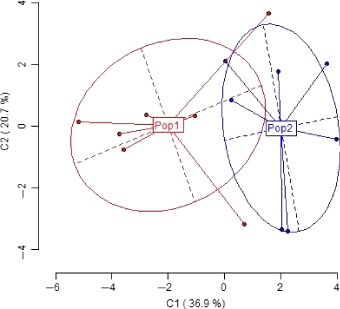
Principal coordinate analysis of methylation‐susceptible loci in the soldiers and the reproductive female castes. Clusters represent the two castes: Pop1 reproductive females, Pop2 soldiers. Explained variance is at 57.6%.

A total of 98 methylation‐susceptible loci (MSL) and 30 nonmethylated loci (NML) were detected. Shannon's diversity index was 0.627 ± 0.083 (mean ± SD) for MSL, while the diversity among NML was 0.607 ± 0.071. Significant differences in diversity between MSL and NML were found (Wilcoxon rank‐sum test with continuity correction: *W *=* *1700.5, *P *=* *0.024). Mean methylation rates in the reproductive females and in the soldiers are reported in Table 2. The difference in methylation frequencies between the castes was not statistically significant (paired *t*‐test: *t*_6_ = 1.075, *P *=* *0.32). Principal coordinate analysis of the MSL revealed significant differences in methylation patterns between the soldiers and reproductive females, as reflected in the associated AMOVA test (*Phi*_ST_ = 0.249, *N* = 14, *P *=* *0.01, Fig. 3). PCA of NML from the two castes, on the other hand, revealed no significant differences between soldiers and reproductive females (associated AMOVA test: *Phi*_ST_ = 0.0347, *N* = 14, *P *=* *0.23).

**Table 2:** Mean ± SD methylation rates in the reproductive females and in the soldiers of Experiment 1. To facilitate comparison with methylation rates reported for other species, global methylation frequencies were calculated in two ways: as the proportion of methylated fragments out of all loci (second data column in the Table) and as the proportion of methylated fragments out of the methylation‐sensitive loci (column 3). Methylation levels of hemimethylated and internal cytosines are reported separately (columns 4‐5), as recommended by Alonso et al. (2016)

Caste	Global (1) (# methylated loci/(# MSL+# NML))	Global (2) (# methylated loci/# MSL)	Hemimethylated cytosines/# MSL	Internal cytosines/# MSL
Reproductive	0.153 ± 0.056	0.199 ± 0.074	0.061 ± 0.025	0.138 ± 0.066
Soldier	0.191 ± 0.109	0.249 ± 0.142	0.171 ± 0.134	0.078 ± 0.034

Of the 27 fragments tested for independence between methylation and caste, only eight showed a single‐sided significance in a Fisher's exact test (i.e., had a single‐sided *P *<* *0.05). There are several theories regarding the presentation of a two‐sided probability in a Fisher's exact test (Agresti 1992). Here, we employed the strictest approach of doubling the single‐sided probability (Table 3). Thus, we can consider each of the 27 Fisher's tests as a Bernoulli trial whose probability of “success” (rejecting the null hypothesis of independence) is 0.10. The probability of at least eight such successes (of 27 trials) is only 3.9 × 10^−3^ (exact binomial test), further indicating differences in methylation patterns between the castes.

**Table 3:** Fragments that were differentially methylated in the reproductive females' caste and the soldier caste. The proportions in the third and fourth columns denote the number of methylated samples, of the seven paired replicates that were analyzed. *P*‐values were obtained by calculating the probabilities for obtaining the observed frequencies at random, using Fisher's exact test. Only methylation patterns with low probability (*P *<* *0.05) of being random are listed. None of the individual *P*‐values lies below the significance threshold, after the Bonferroni correction for multiple testing was applied

		Caste		
Fragment size (bp)	Primer pair	Reproductive females	Soldiers	Pattern significance (*P*‐value)
116	E‐ACA/M‐CT	0/7	5/7	0.0105
124	E‐ACT/M‐CA	0/7	4/7	0.0350
172	E‐ACT/M‐CA	0/7	5/7	0.0105
82	E‐ACC/M‐GA	0/7	4/7	0.0350
92	E‐ACC/M‐GA	0/7	5/7	0.0105
23	E‐ACC/M‐GA	6/7	1/7	0.0146
29	E‐ACC/M‐GA	0/7	4/7	0.0350
242	E‐ACC/M‐GA	4/7	0/7	0.0350

### Experiment 2 – Parental effects on methylation across developmental stages

The experiment generated 120 methylation‐susceptible loci (MSL) and 98 nonmethylated loci (NML) in the 104 samples tested (three primer pairs: E‐ACG/M‐CT, E‐ACT/M‐CA, and E‐ACC/M‐GA). The repeatability scores for methylated loci were 94.5%, 95.77%, and 94.97% for larval (*n* = 2), pupal (*n* = 3), and adult (*n* = 3) samples, respectively. Nonmethylated loci in these samples had repeatability scores of 100% (larvae), 96.75% (pupae), and 98.67% (adults). Shannon's diversity index among MSL was 0.422 ± 0.138, while the mean diversity among NML was only 0.184 ± 0.075. The differences in diversity between MSL and NML were significant (Wilcoxon rank‐sum test with continuity correction: *W *=* *9977, *P *<* *0.0001). The overall percentage of methylated loci was 21.84 ± 2.35%, and average methylation levels for the different experiment groups are reported in Table 4. PCoA of these loci did not reveal significant differences in the composition of methylated fragments among the four crossbreeding combinations (AMOVA test: *Phi*_ST_ = −0.032, *N* = 102, *P *=* *0.989). The two principal axes in the analysis explained 32.4% of the variance of methylated fragments. We also found no significant differences in methylation patterns across the three different developmental stages (AMOVA test: *Phi*_ST_ = −0.017, *N* = 102, *P *=* *0.922). In this analysis, the principal axes explained 32.2% of the variance of methylated fragments.

**Table 4:** Mean methylation rates in the four crossbreeding groups and three developmental stages analyzed in Experiment 2. As in Table 2, global methylation frequencies were calculated in two ways: as the proportion of methylated fragments out of all loci (column Global (1)) and as the proportion of methylated fragments out of the methylation‐sensitive loci (column Global (2)). Methylation levels of hemimethylated and internal cytosines are reported separately in the two rightmost columns

Crossbreeding group	Developmental stage	Global (1) (# methylated loci/(# MSL+# NML))	Global (2) (# methylated loci/# MSL)	Hemimethylated cytosines/# MSL	Internal cytosines/# MSL
♀LD × ♂LD	Larvae	0.103	0.199	0.119	0.079
	Pupae	0.115	0.220	0.132	0.088
	Adults	0.102	0.196	0.083	0.113
♀LD × ♂HD	Larvae	0.137	0.264	0.113	0.150
	Pupae	0.112	0.216	0.116	0.100
	Adults	0.119	0.230	0.108	0.121
♀HD × ♂LD	Larvae	0.114	0.219	0.128	0.091
	Pupae	0.116	0.224	0.131	0.093
	Adults	0.127	0.244	0.129	0.114
♀HD × ♂HD	Larvae	0.098	0.189	0.095	0.094
	Pupae	0.117	0.226	0.112	0.114
	Adults	0.127	0.244	0.135	0.108

**Figure 4:**
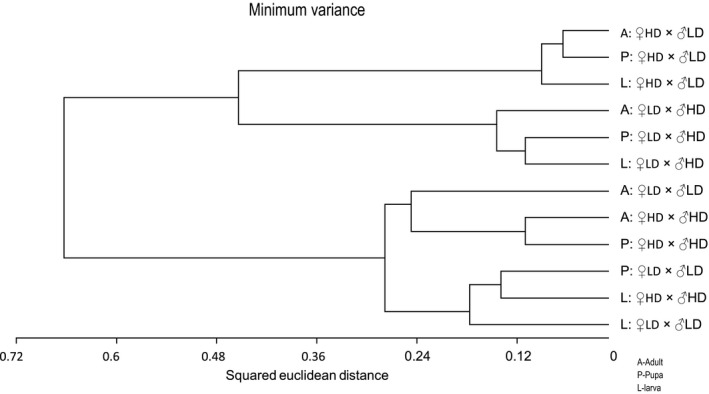
A dendrogram summary of the clustering analysis, which groups the 12 groups of samples (four crossbreeding groups × three developmental stages) by similarity in the composition of hypermethylated fragments. L – larvae, P – pupae, A – adults. The three main clusters are surrounded by dashed frames.

In 31 of the MSL, all samples from one or more of the four crossbreeding groups were not recognized by either endonuclease and thus were considered to be hypermethylated. We paid special attention to these fragments, as they potentially indicate methylations that are shared by individuals subjected to similar parental effects. Table S1 lists the frequencies of hypermethylation for each fragment, for the twelve combinations of crossbreeding group × developmental stage. We characterized each of the 12 experimental groups by a vector with 31 components. Each component is the frequency of a fragment's hypermethylation within the experimental group. Clustering of these vectors by similarity revealed high resemblance in methylation patterns among samples from treatment ♀HD × ♂LD, regardless of developmental stage. Similarly, larvae, pupae, and adults from the ♀LD × ♂HD treatment clustered together. A third cluster contains samples from the two remaining treatments, ♀HD × ♂HD and ♀LD × ♂LD (Fig. 4). To assess the significance of such an assemblage, one can use a combinatorial argument: Altogether we have 12 vectors, three from each treatment. By chance, these can be divided into three groups of 3, 3, and 6 by $\left(\begin{array}{c} 12\\ 3 \end{array}\right)\left(\begin{array}{c} 9\\ 3 \end{array}\right)\left(\begin{array}{c} 6\\ 6 \end{array}\right)=18,480$ different combinations. Only $\left(\begin{array}{c} 4\\ 2 \end{array}\right)=6$ of these exhibit the feature of having a single treatment in each of the two small clusters, and the remaining two treatments in the third cluster. Thus, we have a $\frac{6}{18,480}\approx 3\times 10^{-4}$ probability of obtaining three clusters as in Figure 4 by chance alone.

Nine fragments were hypermethylated in all larval samples, regardless of crossbreeding treatment, and were hypermethylated only in some of the pupal or adult samples. Five were universally hypermethylated in adults but not in all pupae and larvae. Two hypermethylated fragments occurred in all samples of two developmental stages: one in the larval and the pupal stages and one in the pupal and adult stages. No fragment was hypermethylated in all of the pupal samples but only in some samples of the two other stages (Table S2).

## Discussion

This study took advantage of the unique life history of *C. koehleri* to ask whether DNA methylation varies with larval caste (Experiment 1), with parental environment and with the wasps' developmental stage (Experiment 2). In both experiments, the diversity among individuals in MSL was significantly higher than in NML. This finding is compatible with the low number of NML found in the study and with our use of an inbred laboratory population of a clonal species. This implies that the variability among samples in DNA fragment lengths is mostly due to epigenetic factors (different sites being methylated), rather than to genetic variance (differences in DNA sequence). Epigenetic effects thus acted as important correlates of phenotypic differentiation in our study. The overall methylation levels of the studied markers were 22.4 ± 2.4% of MSL in Experiment 1 and 21.84 ± 2.35% in Experiment 2. Although somewhat higher than in previous studies of hymenopterans, these methylation rates are not outstanding: methylation level in bumblebees (*Bombus terrestis*), honeybees (*Apis mellifera*), and other hymenopterans, assessed by MS‐AFLP, ranged from 1% to 19% (Kronforst et al. 2008; Weiner et al. 2013; Amarasinghe et al. 2014).

Methylation levels of the obtained markers in soldiers in Experiment 1 were not significantly higher than in reproductive females. Similarly, no significant differences in overall methylation level between castes were found in *C. floridanus* and *H. saltator* ants (Bonasio et al. 2012), in honeybees and bumblebees, and in a few species of social wasps (Weiner et al. 2013). While similar in overall methylation levels, *C. koehleri* soldiers and reproductives differed significantly in the composition of methylated DNA fragments. A few fragments were methylated in one caste but not in the other. This is compatible with the finding that six odorant‐binding protein genes are differentially expressed in soldiers vs. reproductives of *Copidosoma floridanum* (Donnell 2014). A similar picture was found in honeybees, where about 10% of the methylated genes showed significant differences in the extent of methylation between queens and workers (Lyko et al. 2010). The pattern of methylated fragments also differs significantly between castes in the primitively social wasp *Polistes dominula*, but not in eusocial wasps and bees tested in the same study (Weiner et al. 2013). Our results, combined with these previous studies, are congruous with a role for differential methylation in caste formation of both social and nonsocial Hymenoptera.

The standard analysis of Experiment 2 does not suggest parental epigenetic effects, that is, effects of parental rearing conditions on DNA methylation in their offspring. The frequency of methylated fragments did not differ among crossbreeding groups, and PCoA analysis revealed no significant differences in their composition. Can parental effects consequently be ruled out? A finer analysis, which considered only the subset of the fragments that were hypermethylated in all samples of a crossbreeding group, provides some evidence to the contrary. In this analysis, methylated samples of the crossbreeding group ♀HD × ♂LD clustered together, as did the samples originating from the reciprocal cross ♀LD × ♂HD. This indicates that larvae, pupae, and adult offspring originating from each of these crosses have similar DNA methylation patterns. The samples from the two remaining crosses, ♀HD × ♂HD and ♀LD × ♂LD, formed a third and different cluster. That is, offspring of these crosses do not vary much in methylation patterns, but differ from the offspring of the crosses ♀HD × ♂LD and ♀LD × ♂HD (Fig. 4). Possibly, the parental epigenetic marks were more effectively transmitted to offspring in the groups ♀HD × ♂LD and ♀LD × ♂HD (so that each group retained a characteristic methylation pattern) than in the groups ♀HD × ♂HD/♀LD × ♂LD. This possibility cannot be tested with our present data, as we did not characterize the methylation profiles of the parent generation. Yet, it is compatible with the recent demonstration that matrigenes and patrigenes are differentially expressed during colony development in honeybees (Galbraith et al. 2016).

The detailed analysis of Experiment 2 also revealed hypermethylated fragments that were shared by all samples of one developmental stage but occurred only in some samples of the other stages. Some hypermethylated fragments occurred in all larval samples, and others appeared in all adult samples. No methylated fragments occurred in all pupae, but only in some larvae and adults. This is probably because during the pupal stage, individuals go through metamorphosis, resulting in considerable changes in methylation patterns. Shared methylated fragments were found in all larvae and pupae, and others occurred in all pupae and adults, suggesting that some methylation patterns change gradually during the pupal stage as individuals develop from larvae into adult. These dynamics correspond with methylation dynamics in bees and ants, where tens to hundreds of differentially methylated genes differ between larvae and adult individuals (Lyko et al. 2010; Bonasio et al. 2012). We were unable to detect “universal” methylated fragments, namely fragments that appeared in all crossbreeding groups at all life stage. Nevertheless, the clustering of methylated fragments by crossbreeding groups hints that environmental cues can leave stable marks across the life cycle, in spite of massive reprogramming during the pupal stage.

The MS‐AFLP technique used in this study is inferior to bisulfite sequencing in detecting far fewer methylated sites and in providing no information on their sequence and function. Therefore, the correlations between the methylation patterns observed in our study and their phenotypic effects are yet to be elucidated. MS‐AFLP is nevertheless considered a powerful technique to investigate the diversity of DNA methylations in species with no reference sequenced genome (Alonso et al. 2016), for example, for comparing among ecotypes within a species (Nicotra et al. 2015). Being relatively rapid and inexpensive, this technique allowed us to analyze 7–10 replicate samples of each crossbreeding group, developmental stage, and caste. This enabled us to look for methylation patterns that were shared between the different samples. The replicated design of our study improves on some previous investigations of DNA methylation in social insects, which analyzed a single colony and thus could not avoid source‐colony effects on methylation patterns (Libbrecht et al. 2016). Additional well‐replicated studies, combined with experimental manipulations of specific methylated genes, are needed to elucidate the role of DNA methylation in phenotypic plasticity in insects.

## Conflict of Interest

None declared.

## Supporting information


**Table S1.** Frequencies of hyper‐methylated fragments, grouped by crossbreeding group.
**Table S2.** Frequencies of hyper‐methylated fragments, grouped by developmental stage.
