# Supplementary material for: Genome methylation patterns across castes and generations in a parasitoid wasp
Source: Ecol Evol. 2016 Sep 30;6(22):7943–53. doi: 10.1002/ece3.2395 (PMC5108247; doi:10.1002/ece3.2395)
Supplement: Supplementary file 1 — Table S1. Frequencies of hyper‐methylated fragments, grouped by crossbreeding group. Table S2. Frequencies of hyper‐methylated fragments, grouped by developmental stage. [file ECE3-6-7943-s001.docx]

Appendix:

Table S1: Frequencies of hyper-methylated fragments, grouped by crossbreeding group. Shaded cells denote fragments that were hyper-methylated in all samples of at least one crossbreeding group.

| **Life**  **Stage** |  | **Larvae** | | | | **Pupae** | | | | **Adults** | | | |
| --- | --- | --- | --- | --- | --- | --- | --- | --- | --- | --- | --- | --- | --- |
| **Fragment length (bp)** | **Primers** | ♀LD  X  ♂LD | ♀LD  X  ♂HD | ♀HD  X  ♂LD | ♀HD  X  ♂HD | ♀LD  X  ♂LD | ♀LD  X  ♂HD | ♀HD  X  ♂LD | ♀HD  x  ♂HD | ♀LD  X  ♂LD | ♀LD  X  ♂HD | ♀HD  X  ♂LD | ♀HD  X  ♂HD |
| 63 | E-ACA/ M-CT | 1 | 0.943 | 1 | 1 | 1 | 0.953 | 0.949 | 1 | 1 | 0.943 | 0.926 | 0.935 |
| 65 | E-ACA/ M-CT | 0.655 | 1 | 1 | 1 | 0.791 | 1 | 0.882 | 0.943 | 0.894 | 1 | 0.866 | 0.882 |
| 66 | E-ACA/ M-CT | 0.857 | 0.889 | 1 | 0.875 | 1 | 1 | 1 | 1 | 1 | 0.889 | 1 | 1 |
| 69 | E-ACA/ M-CT | 1 | 0.889 | 1 | 1 | 1 | 0.9 | 0.9 | 1 | 0.857 | 1 | 1 | 1 |
| 70 | E-ACA/ M-CT | 1 | 0.943 | 1 | 1 | 1 | 0.953 | 1 | 0.949 | 1 | 0.943 | 1 | 0.943 |
| 75 | E-ACA/ M-CT | 1 | 1 | 1 | 1 | 1 | 1 | 1 | 1 | 1 | 0.889 | 0.875 | 0.875 |
| 76 | E-ACA/ M-CT | 1 | 1 | 1 | 0.875 | 0.857 | 1 | 0.875 | 0.778 | 1 | 1 | 1 | 1 |
| 80 | E-ACA/ M-CT | 1 | 1 | 1 | 0.75 | 0.714 | 1 | 1 | 0.778 | 0.5 | 1 | 1 | 0.667 |
| 81 | E-ACA/ M-CT | 0.857 | 0.857 | 1 | 0.833 | 0.714 | 0.875 | 1 | 1 | 1 | 0.857 | 1 | 0.875 |
| 87 | E-ACA/  M-CT | 1 | 1 | 1 | 1 | 1 | 0.909 | 0.889 | 0.9 | 1 | 1 | 1 | 0.889 |
| 63 | E-ACG/  M-CA | 1 | 0.75 | 1 | 1 | 0.857 | 0.8 | 0.778 | 1 | 1 | 0.875 | 0.875 | 1 |
| 73 | E-ACG/ M-CA | 1 | 1 | 1 | 1 | 1 | 1 | 0.9 | 1 | 0.714 | 1 | 0.875 | 1 |
| 76 | E-ACG/ M-CA | 1 | 1 | 0.889 | 0.875 | 1 | 1 | 0.9 | 0.9 | 1 | 0.778 | 1 | 0.778 |
| 83 | E-ACG/  M-CA | 0.857 | 1 | 0.889 | 1 | 1 | 1 | 1 | 1 | 0.857 | 1 | 1 | 1 |
| 87 | E-ACG/  M-CA | 0.857 | 1 | 1 | 1 | 0.889 | 0.909 | 0.889 | 1 | 1 | 1 | 1 | 1 |
| 88 | E-ACG/  M-CA | 0.857 | 1 | 0.889 | 1 | 1 | 0.909 | 1 | 1 | 1 | 1 | 1 | 1 |
| 100 | E-ACG/  M-CA | 0.857 | 0.889 | 1 | 1 | 0.889 | 1 | 1 | 0.9 | 1 | 1 | 1 | 0.875 |
| 106 | E-ACG/  M-CA | 1 | 0.889 | 0.889 | 1 | 1 | 1 | 1 | 0.9 | 1 | 1 | 1 | 0.889 |
| 107 | E-ACG/  M-CA | 1 | 0.889 | 1 | 1 | 0.889 | 0.909 | 1 | 1 | 0.857 | 0.889 | 1 | 1 |
| 121 | E-ACG/  M-CA | 1 | 1 | 0.778 | 1 | 1 | 1 | 0.8 | 1 | 1 | 1 | 0.875 | 1 |
| 175 | E-ACG/  M-CA | 0.857 | 1 | 1 | 1 | 0.778 | 0.909 | 1 | 0.9 | 0.857 | 1 | 1 | 0.75 |
| 211 | E-ACG/  M-CA | 1 | 0.444 | 1 | 1 | 0.778 | 0.818 | 1 | 1 | 1 | 0.778 | 1 | 0.778 |
| 212 | E-ACG/  M-CA | 1 | 1 | 0.778 | 1 | 0.889 | 1 | 0.8 | 0.8 | 0.857 | 1 | 0.875 | 0.889 |
| 253 | E-ACG/ M-CA | 1 | 1 | 0.889 | 1 | 1 | 1 | 0.9 | 0.9 | 1 | 1 | 1 | 0.875 |
| 265 | E-ACG/ M-CA | 1 | 0.889 | 1 | 0.875 | 1 | 0.909 | 1 | 1 | 1 | 1 | 1 | 0.889 |
| 68 | E-ACC/ M-GA | 1 | 1 | 0.889 | 0.875 | 0.889 | 1 | 0.9 | 1 | 0.857 | 1 | 0.875 | 1 |
| 78 | E-ACC/ M-GA | 1 | 1 | 1 | 1 | 1 | 1 | 1 | 0.9 | 1 | 0.778 | 1 | 0.889 |
| 100 | E-ACC/ M-GA | 0.857 | 0.889 | 1 | 0.857 | 0.889 | 0.818 | 1 | 1 | 0.857 | 1 | 1 | 1 |
| 141 | E-ACC/ M-GA | 0.667 | 0.875 | 1 | 0.625 | 0.667 | 0.9 | 1 | 0.9 | 0.714 | 1 | 1 | 0.75 |
| 147 | E-ACC/ M-GA | 0.857 | 1 | 0.889 | 0.857 | 0.889 | 1 | 1 | 1 | 0.857 | 1 | 0.875 | 1 |
| 272 | E-ACC/ M-GA | 1 | 1 | 0.889 | 1 | 1 | 1 | 0.8 | 1 | 1 | 1 | 0.875 | 1 |

Table S2: Frequencies of hyper-methylated fragments, grouped by developmental stage. Shaded cells denote fragments that were hyper-methylated in all samples.

|  | | **Developmental stage** | | |
| --- | --- | --- | --- | --- |
| **Fragment length (bp)** | **Primer** | **Larva** | **Pupa** | **Adult** |
| **60** | E-ACA/M-CT | 32/32 | 13/38 | 18/34 |
| **77** | E-ACA/M-CT | 32/32 | 22/38 | 7/34 |
| **79** | E-ACA/M-CT | 20/32 | 21/38 | 34/34 |
| **87** | E-ACA/M-CT | 32/32 | 12/38 | 26/34 |
| **75** | E-ACG/M-CA | 32/32 | 13/38 | 19/34 |
| **96** | E-ACG/M-CA | 32/32 | 5/38 | 19/34 |
| **98** | E-ACG/M-CA | 32/32 | 21/38 | 14/34 |
| **102** | E-ACG/M-CA | 25/32 | 13/38 | 34/34 |
| **124** | E-ACG/M-CA | 18/32 | 29/38 | 34/34 |
| **141** | E-ACG/M-CA | 18/32 | 29/38 | 34/34 |
| **181** | E-ACG/M-CA | 32/32 | 38/38 | 19/34 |
| **189** | E-ACG/M-CA | 32/32 | 21/38 | 19/34 |
| **196** | E-ACG/M-CA | 32/32 | 30/38 | 12/34 |
| **70** | E-ACC/M-GA | 13/32 | 30/38 | 34/34 |
| **99** | E-ACC/M-GA | 32/32 | 15/38 | 20/34 |
| **140** | E-ACC/M-GA | 32/32 | 13/38 | 26/34 |
| **215** | E-ACC/M-GA | 10/32 | 38/38 | 34/34 |
